# Accurate identification of snoRNA targets using variational graph autoencoder to advance the redevelopment of traditional medicines

**DOI:** 10.3389/fphar.2024.1529128

**Published:** 2025-01-06

**Authors:** Zhina Wang, Yangyuan Chen, Hongming Ma, Hong Gao, Yangbin Zhu, Hongwu Wang, Nan Zhang

**Affiliations:** ^1^ Department of Pulmonary and Critical Care Medicine II, Emergency General Hospital, Beijing, China; ^2^ Department of Oncology, Emergency General Hospital, Beijing, China; ^3^ School of Data Science and Artificial Intelligence, Wenzhou University of Technology, Wenzhou, China; ^4^ Respiratory Disease Center, Dongzhimen Hospital, Beijing University of Chinese Medicine, Beijing, China

**Keywords:** redevelopment of traditional medicines, snoRNA therapeutic targets, lung cancer, variational graph autoencoder (VGAE), artificial intelligence (AI)

## Abstract

Existing studies indicate that dysregulation or abnormal expression of small nucleolar RNA (snoRNA) is closely associated with various diseases, including lung cancer. Furthermore, these diseases often involve multiple targets, making the redevelopment of traditional medicines highly promising. Accurate prediction of potential snoRNA therapeutic targets is essential for early disease intervention and the redevelopment of traditional medicines. Additionally, researchers have developed artificial intelligence (AI)-based methods to screen and predict potential snoRNA therapeutic targets, thereby advancing traditional drug redevelopment. However, existing methods face challenges such as imbalanced datasets and the dominance of high-degree nodes in graph neural networks (GNNs), which compromise the accuracy of node representations. To address these challenges, we propose an AI model based on variational graph autoencoders (VGAEs) that integrates decoupling and Kolmogorov-Arnold Network (KAN) technologies. The model reconstructs snoRNA-disease graphs by learning snoRNA and disease representations, accurately identifying potential snoRNA therapeutic targets. By decoupling similarity from node degree, the model mitigates the dominance of high-degree nodes, enhances prediction accuracy in scenarios like lung cancer, and leverages KAN technology to improve adaptability and flexibility to new data. Case studies revealed that snoRNA SNORA21 and SNORD33 are abnormally expressed in lung cancer patients and are strong candidates for potential therapeutic targets. These findings validate the proposed model’s effectiveness in identifying therapeutic targets for diseases like lung cancer, supporting early screening and treatment, and advancing the redevelopment of traditional medicines. Data and experimental findings are archived in: https://github.com/shmildsj/data.

## Introduction

SnoRNA is a type of non-coding RNA, typically 60 to 300 nucleotides long, and predominantly found in eukaryotic cells (Esteller, 2011). Based on function, snoRNAs are categorized into two types: box C/D and box H/ACA (Liao et al., 2010). Numerous studies have demonstrated that snoRNA plays a crucial regulatory role. For instance, snoRNA regulates the methylation of rRNA and tRNA (Kiss-László et al., 1996), and is also involved in mRNA splicing (Kishore and Stamm, 2006). SnoRNA can bind to specific proteins to prevent enzyme cleavage and facilitate related RNA processing (Esteller, 2011). Furthermore, growing evidence suggests that snoRNA is a key factor in the occurrence and progression of various diseases. For example, dysregulation of snoRNA may promote various diseases, including gastric cancer (Wang et al., 2019). SnoRNA downregulation may lead to brain cavernous malformations (Qi et al., 2016), and promote lung cancer (Liao et al., 2010) or tumor development (Krishnan et al., 2016). Similarly, a significant increase in snoRNA levels may promote the progression of various cancers (Zheng et al., 2015). Thus, studying the regulatory mechanisms of snoRNA is crucial for advancing disease treatment strategies. Clinical trials can help accurately uncover the regulatory mechanisms of specific snoRNAs in diseases. However, these methods often depend on long-term experiments and observations, as well as costly equipment.

Fortunately, previous research has accumulated substantial data, enabling data scientists to uncover new regulatory mechanisms of snoRNA in diseases. For instance, X et al. developed the MNDR database, which is based on the regulatory mechanisms of mammalian ncRNA in diseases (Ning et al., 2021). Chen and Zhang et al. integrated and updated the RNA-disease association databases RNADisease v4.0 and ncRPheno, making them publicly accessible (Chen et al., 2023; Zhang W. et al., 2020). These databases contain SDA data and have the potential to advance the development of related computational methods. For example, Sun et al. collected high-quality SDA data from the MNDR database, integrated snoRNA and disease similarity networks, and predicted unknown SDAs using matrix completion (Sun et al., 2022). Hu et al. gathered known SDAs from RNADisease v4.0 and ncRPheno, constructed snoRNA-disease networks, and applied subgraph extraction and graph collaborative filtering to identify unknown SDAs (Hu et al., 2024). Overall, relatively few computational methods directly explore SDAs. And these models depend on complex feature extraction and classification processes, which significantly limit their broad applicability. However, numerous studies focus on potential interactions in biological networks, which are fundamentally similar to SDA prediction tasks. Advanced methods primarily include deep learning, GNNs, and graph autoencoder techniques.

The rapid development of deep learning is evident in its widespread application in bioinformatics (Wang Y. et al., 2023; Wang R. et al., 2024; Wang R. et al., 2023; Zhuo et al., 2023), particularly in exploring potential interactions within biological networks, achieving notable success. For instance, Zhou et al. automatically extracted sequence features of lncRNA and proteins using the Transformer architecture, integrated them to obtain representations of lncRNA-protein pairs, and predicted unknown lncRNA-protein interactions via multi-layer perceptrons (Zhou et al., 2023). Wei et al. incorporated data augmentation, feature alignment, and other techniques into a self-supervised learning framework, extracting precise node representations to efficiently predict unknown food-drug interactions (Wei et al., 2024a). Liu et al. applied meta-path technology to extract features of circular RNA and diseases, integrated them to obtain representations of circular RNA-disease pairs, and predicted unknown circular RNA-disease associations using contrastive learning and MLP (Liu et al., 2023a). Then, Liu et al. proposed a novel method for predicting gene regulatory networks, refining the topological network from both global and local perspectives, and accounting for edge importance to enhance prediction performance (Liu et al., 2023b). Additionally, Wei et al. employed an integrated deep learning framework, incorporating clustering-based parameter fine-tuning, to significantly enhance the accuracy of drug-target interaction prediction (Wei et al., 2024b). Moreover, Wei demonstrated that large language models have a significant impact on drug repositioning (Wei et al., 2024c). Deep learning technology effectively extracts deep node representations, operates independently of manually designed features, and swiftly and accurately infers potential interactions within biological networks. However, a major drawback of these methods is their neglect of the topological information in known biological networks.

Graph neural networks (GNNs) uncover the structure of topological networks via message propagation mechanisms. GNNs have emerged in bioinformatics fields like property prediction (Wang et al., 2024b; Ma et al., 2024a; Wang et al., 2024c) and gene detection (Ma et al., 2024b; Wang et al., 2024d), challenging traditional machine learning and deep learning approaches. Similarly, GNN technologies play a crucial role in predicting interactions within biological networks. For example, Zhuo et al. used two graph convolutional networks to extract node representations of lncRNA and proteins, followed by pairwise learning to train predictors for identifying unknown lncRNA-protein interactions (Zhuo et al., 2022). Wei et al. subsequently applied sampling to enhance the GCN model’s performance in handling sparse data (Wei et al., 2023a). Liao et al. employed an autoencoder to extract representations of miRNA and diseases and used GCN to predict unknown miRNA-disease associations (Liao et al., 2023). Wei et al. applied a graph collaborative filtering model and multi-perspective contrastive learning to enhance node representations, aiming to accurately predict unknown miRNA-drug sensitivity (Wei et al., 2023b). Furthermore, Zhou et al. noted that message propagation across the entire graph may lead to “over-smoothing” and adopted a subgraph enhancement strategy to improve microbial-drug interaction predictions by focusing on local features (Zhou et al., 2024a). Building on this, Zhou et al. adopted an energy-constrained diffusion mechanism to extract global node representations of drugs and proteins, uncovering potential drug-protein relationships and accurately predicting unknown interactions (Zhou et al., 2024b). Additionally, Li et al. integrated multi-source similarity networks of miRNA and diseases to enhance model performance in predicting miRNA-disease interactions (Li Z. et al., 2024). GNN technology explores the topological information of biological networks via message propagation mechanisms, significantly enhancing interaction analysis within these networks. However, methods related to GNN often overlook the intrinsic information of the nodes. Additionally, the dominance of high-degree nodes in message propagation within GNNs can limit model performance.

The integration of self-supervised learning strategies with GNN technology has driven the development of graph autoencoders, widely applied to uncover potential information in biological networks. For instance, Zhou et al. proposed a method utilizing the graph autoencoder framework and edge masking to predict potential interactions between small molecules and miRNAs (Zhou et al., 2024c). This method first masks edges in the small molecule-miRNA graph using a Bernoulli distribution, then applies a GNN encoder and inner product decoder to reconstruct the masked graph. Zhou et al. subsequently masked paths in the miRNA-drug graph and trained the miRNA-drug association predictor using graph autoencoder technology (Zhou et al., 2024d). Zhang et al. subsequently integrated multi-source similarity networks and employed graph autoencoders along with polyloss technology to infer unknown lncRNA-protein interactions (Zhang et al., 2024a). Graph autoencoders are becoming increasingly crucial in biological network research due to their self-supervised nature, simplicity, and efficiency. Like GNN models, GAE-related models also encounter issues with message propagation dominated by high-degree nodes.

In summary, these advanced models have demonstrated success in interaction prediction within biological networks and should be capable of handling snoRNA-disease prediction tasks. However, these models face several significant challenges. First, these models, particularly graph-based models, have an inherent limitation due to their message propagation mechanism. Studies have shown that nodes with high degrees tend to have large embedding norms, which dominate message propagation and hinder GNN models from extracting accurate snoRNA or disease representations. Second, extremely sparse or isolated snoRNA or disease nodes often exist in the snoRNA-disease graph, leading to unpredictable results during gradient backpropagation. To address these issues, we incorporate L2 regularization and decoupling techniques into the variational graph autoencoder framework, proposing a snoRNA-disease association prediction model, named DK-SDA, to mitigate these challenges and enhance prediction performance. Our contributions can be summarized as follows:

(1) We proposed an SDA prediction model within the VGAE framework, yielding reliable results.
(2) We employed graph-regularized convolutional networks to extract snoRNA and disease representations, mitigating the issue of sparse nodes causing unpredictable gradient propagation.
(3) We applied decoupling technology to separate snoRNA-disease similarity from the node degrees of snoRNA or disease, thus mitigating the dominant effect of high-degree nodes on message propagation.
(4) We conducted multiple experiments on public datasets, and the results confirmed the model’s high efficiency in lung cancer research, providing strong support for early screening and treatment.

## Materials and methods

### Data preparation

This study utilized two datasets collected by previous research (Hu et al., 2024) to evaluate the performance of the proposed and comparison models. The first dataset was sourced from the RNADisease v4.0 (Chen et al., 2023) database, where experimentally verified and predicted SDAs were extracted, with duplicates and missing data removed. The final dataset included 471 snoRNAs, 84 diseases, and 1,095 SDAs. The dataset was then divided into training and test sets in a 4:1 ratio. The second dataset was sourced from the ncRPheo database (Zhang W. et al., 2020). After data cleaning, it contained 82 diseases, 13 snoRNAs, and 439 SDAs. This dataset was used as an external test set to evaluate model performance under isolation conditions.

### Method

This study proposes a snoRNA-disease association prediction model within the VGAE framework, aiming to efficiently predict unknown snoRNA-disease pairs from observed SDAs. Compared to other advanced interaction prediction models in biological networks, this model presents two key differences. First, the model addresses sparse nodes in the snoRNA-disease network, which can undermine the reliability of the extracted embeddings. Then, L2 regularization is applied to process node representations in both the initial and intermediate layers to alleviate the difficulty in predicting low-degree node embeddings during gradient backpropagation. Second, the model addresses the dominant effect of high-degree nodes on message propagation. Therefore, decoupling technology is introduced to separate snoRNA-disease similarity from node norms, thereby enhancing the reliability of message propagation. The following sections will elaborate on the relevant principles and techniques.

#### Preliminary

In this study, the snoRNA-disease network is represented as ***G = <V,E,X>***, where ***V = Vs*∪*Vd*** denotes the set of snoRNA and disease nodes, ***E∈Vs × Vd*** denotes the observed SDAs, and ***X = Xs*∪*Xd*** denotes the initial feature set of snoRNA and disease nodes. A graph convolutional network (GCN) is used as an encoder within the VGAE framework to perform operations such as message propagation and node embedding extraction. Recently, studies have focused on the L2 norm of node embeddings across various fields. For example, in translation, uncommon words are often assigned lower L2 norms (Kobayashi et al., 2020). In image processing or computer vision, low-quality images tend to have lower L2 norms for their corresponding embeddings (Liu et al., 2017). Subsequent studies have employed L2 regularization to reduce quantization error between large and small norms (Ranjan et al., 2017). Zhang et al. noted that L2 regularization can alleviate the instability between large and small norms during gradient backpropagation (Zhang D. et al., 2020). Additionally, Zheng et al. have applied L2 regularization in GCNs to mitigate the over-smoothing problem during message propagation (Zheng et al., 2018).

#### Model framework

**FIGURE 1:**
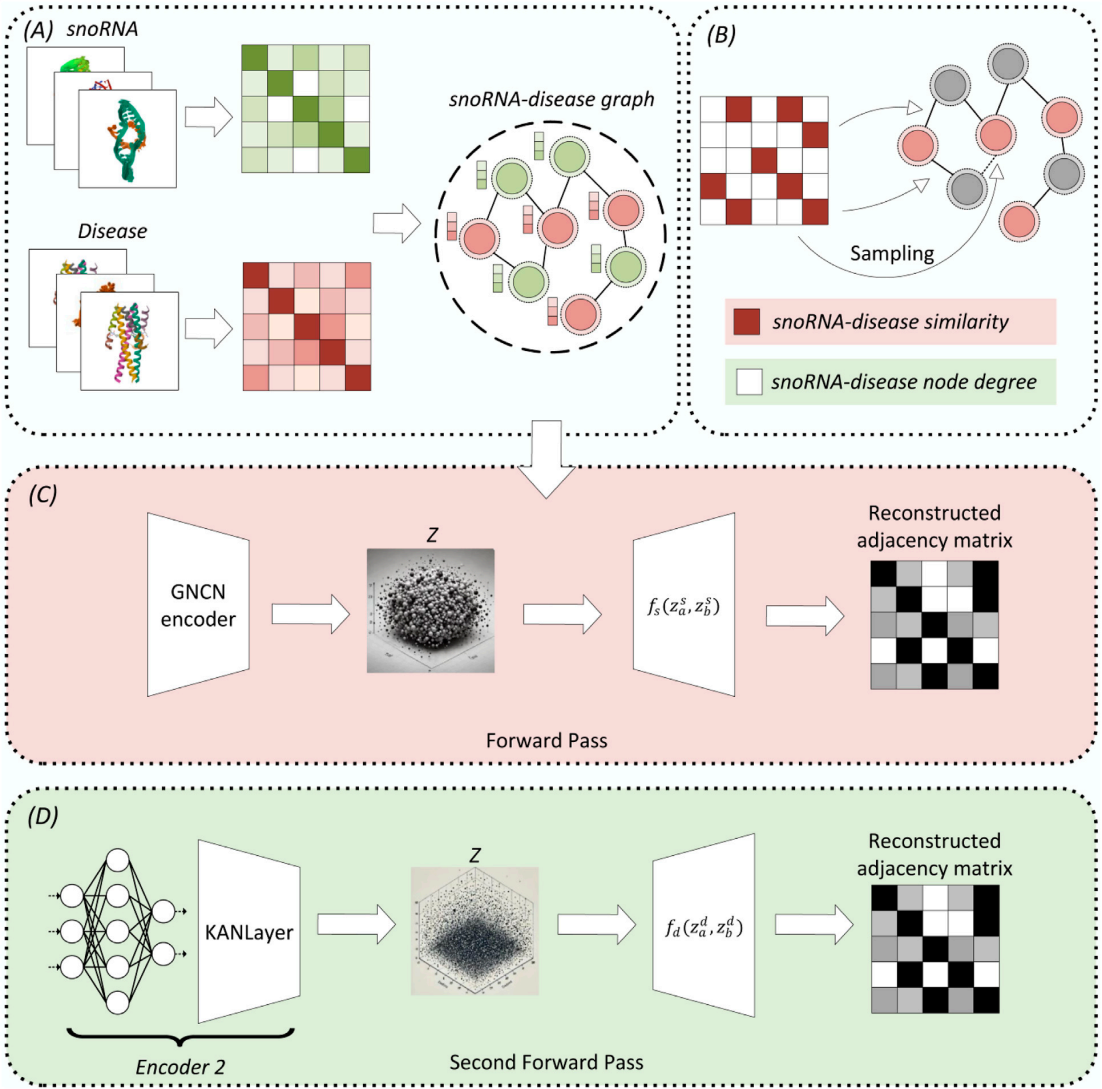
DK-SDA model’s architecture, comprising: **(A)** construction of the snoRNA-disease network, **(B)** the sampling process, **(C)** reconstruction of the adjacency matrix based on snoRNA-disease similarity, and **(D)** reconstruction of the adjacency matrix based on node degree.

Figure 1 depicts the DK-SDA model’s architecture, which encompasses (A) snoRNA-disease network construction, (B) sampling process (C) adjacency matrix reconstruction based on snoRNA-disease similarity, and (D) adjacency matrix reconstruction based on node degree. In Module A, data retrieved from the database constructs the snoRNA-disease network. Module C initiates with Bernoulli sampling as per Module B. If the SDAs are established based on snoRNA-disease similarity, the process concludes; otherwise, it transitions to Module D for adjacency matrix reconstruction based on node degree.

#### L2-normalization in GCN

To mitigate the issue of unpredictable gradient propagation caused by sparse nodes, we introduced L2 regularization in GCN based on previous work (Ahn and Kim, 2021). Specifically, L2 regularization is applied before GCN performs message propagation. For a snoRNA or disease node ***a***, its initial feature is represented as ***x***_***a***_, which is mapped to a trainable weight matrix as:

$$h_{a}={g\left(x_{a}W\right)=x}_{a}W,$$ (1)

Next, L2 regularization is applied to ***h***_***a***_ as defined in Equation 2:

$$n_{a}=t\frac{h_{a}}{\left\|h_{a}\right\|},$$ (2)

where ***t*** represents the scaling parameter, set to 1.8 by default. Based on the regularized representation, GCN performs message propagation and outputs the embedding of node ***a***:

$$z_{a}=\frac{1}{d_{a}+1}n_{a}+\sum_{b\in Na}\frac{1}{\sqrt{d_{a}+1\sqrt{d_{b}+1}}}n_{b},$$ (3)

where Na represents the set of neighbor nodes of node ***a***, and $d_{a}$ represents the degree of node ***a***. Notably, in the subsequent process, the similarity between snoRNA and disease and the norms of snoRNA or disease nodes are decoupled. The similarity and node norms are sampled from the embeddings extracted by GCN, and the GCN corresponding to the node norm does not execute Equation 3. Then the operation of this GCN can be formalized as Equation 4:

$$GNCN\left(X,A,t\right)=t{\hat{D}}^{-\frac{1}{2}}\hat{A}{\hat{D}}^{-\frac{1}{2}}g\left(XW\right),$$ (4)

where $\hat{D}$ represents the degree matrix and $\hat{A}$ represents the adjacency matrix with self-loop.

#### Variational graph autoencoder (VGAE) framework

Generally, the node embedding matrix ***Z*** output by GCN can be used with an inner product decoder to reconstruct the snoRNA-disease network $A=g\left(ZZ^{T}\right)$ . However, studies suggest that variational graph autoencoders (VGAE) (Kipf and Welling, 2016) offer greater advantages. The implementation of VGAE is straightforward, consisting primarily of two components: a GNN encoder and a decoder. The GCN encoder serves as an inference model, sampling and deriving the variational posterior of node embeddings based on an approximate Gaussian distribution. The snoRNA-disease network is then reconstructed using the decoder. In the GCN encoder, the posterior of node embeddings is difficult to derive directly and is typically approximated using a Gaussian distribution (Kipf and Welling, 2016) as Equation 5:

$$q\left(Z|A,X\right)=\prod_{a=1}^{n}q\left(z_{a}\left|A,X\right.\right),$$ (5)

where

$$q\left(z_{a}\left|A,X\right.\right)=N\left(z_{a}|\mu_{a},diag\left(\sigma_{a}^{2}\right)\right),$$ (6)

and

$$\mu_{a}={GNCN}_{\mu_{a}}\left(A,X,t\right),\sigma_{a}={GCN}_{\sigma_{a}}\left(A,X\right).$$ (7)

In Equations 6 and 7, $\mu_{a}$ and $\sigma_{a}$ share the first layer parameters. And $\mu_{a}$ undergoes regularization, while $\sigma_{a}$ does not. Both $\mu_{a}$ and $\sigma_{a}$ perform the propagation process independently. Equation 7 are used for feature extraction based on snoRNA-disease similarity. This study also incorporates an encoder based on node degree. However, unlike Equation 7, the mean and variance based on node degree are calculated without applying Equation 3.

#### Kolmogorov-Arnold Network (KAN)

The Multilayer Perceptron (MLP) has been instrumental in the field of deep learning due to its strong ability to approximate nonlinear functions. However, MLP faces unavoidable challenges, including large parameter sizes and limited flexibility. To address these challenges, KAN technology was developed (Liu et al., 2024). The core concept of the Kolmogorov-Arnold representation theorem is that any multivariate continuous function can be expressed as a composition of continuous univariate functions:

$$f\left(h_{1},\ldots ,h_{n}\right)=\sum_{v=1}^{2n+1}\pi_{v}\left(\sum_{u=1}^{n}\omega_{v,u}\left(h_{u}\right)\right),$$ (8)

In Equation 8 theorem establishes the theoretical foundation that any multivariate continuous function can be represented as the sum of continuous univariate functions. KAN technology leverages this theory to efficiently approximate complex functions. In this framework, $\omega_{v,u}\left(\;\right)$ functions as a univariate transformation of each input variable $h_{u}$ , where $\omega_{v,u}:\left[0,1\right]\to R$ and $\pi_{v}:R\to R$ . Given that all functions are univariate, each can be parameterized as b-spline curves with local basis function coefficients that can be optimized.

Building on the structure of the multi-layer perceptron (MLP), a deeper KAN architecture is constructed by stacking ***L*** layers:

$$KAN\left(h\right)=\left(\Theta_{L}\circ \Theta_{L-1}\circ \cdots \circ \Theta_{1}\right)h,$$ (9)

where $\Theta_{i}$ denotes the parameters of the ***i***-th layer. Therefore, KAN can externally stack multiple layers, similar to MLP, to extract deep features as Equation 9. Internally, the univariate function within KAN offers greater flexibility to the model. This study employs the KAN network architecture in the second layer of the node degree-based encoder.

KANs offer a novel approach that enhances flexibility and interpretability compared to traditional MLPs by using spline functions as edge weights. This design allows KANs to better capture the underlying features driving snoRNA-disease interactions, thereby enhancing the model’s interpretability. Researchers can analyze the spline function coefficients to understand how the network processes input data and makes predictions, making KANs a more transparent alternative to other neural network architectures.

#### Decoupling node similarity and degree

In typical VGAE-based methods, message propagation relies on both node similarity and norm. When these two factors are misaligned, the norm often dominates message propagation. Several studies have shown that in networks or graphs, the embedding norm of low-degree nodes is typically low, while high-degree nodes have higher norms (Cho, 2024). According to prior studies (Cho, 2024), there are two conditions under which snoRNA and disease can be considered associated. The first condition is a high degree of association between snoRNA and disease, indicated by high similarity. This is referred to as snoRNA-disease similarity. The second condition is when a snoRNA or disease node has a high degree, indicating its popularity, which increases the likelihood of forming associations with other nodes, though they may remain undiscovered. This is referred to as node degree. However, when establishing an association between snoRNA and disease, it is difficult to determine which condition applies due to the lack of precise prior knowledge. Therefore, for each snoRNA-disease pair, this study employs Bernoulli distribution sampling based on snoRNA-disease similarity to determine which condition applies for establishing SDAs.

Therefore, this study employed the previously established method (Cho, 2024), extracting node embeddings based on snoRNA-disease similarity and node degree, followed by constructing individual decoders. For snoRNA ***a*** or disease ***b***, sampling is conducted based on similarity to derive the potential node embedding as Equation 10:

$$z_{a}^{s}∼N\left(u^{s},\sigma^{s}\right),z_{b}^{s}∼N\left(u^{s},\sigma^{s}\right),$$ (10)

where $u^{s}$ and $\sigma^{s}$ are calculated using Equation 7. For snoRNA ***a*** or disease ***b***, sampling is conducted based on node degree to derive the potential node embedding as Equation 11:

$$z_{a}^{d}∼N\left(u^{d},\sigma^{d}\right),z_{b}^{d}∼N\left(u^{d},\sigma^{d}\right),$$ (11)

where $u^{d}$ and $\sigma^{d}$ are calculated using Equation 7. Notably, Equation 3 is not applied at this stage.

For any snoRNA-disease pair ***<a,b>***, a two-stage approach is employed to establish their association:

(1) SnoRNA-disease similarity: Establish SDAs based on the Bernoulli distribution.

$$A_{a,b}∼Bernoulli\left(\sigma \left(f_{s}\left(z_{a}^{s},z_{b}^{s}\right)\right)\right),$$ (12)

where σ represents the SIGMOD function, $f_{s}$ **()** measures the similarity between snoRNA and disease, and the normalized inner product can be used.

(2) Node Degree: If no association is established after the initial sampling, continue sampling as follows.

$$A_{a,b}∼Bernoulli\left(\sigma \left(f_{d}\left(z_{a}^{d},z_{b}^{d}\right)\right)\right),$$ (13)

where $f_{d}$ **()** measures node popularity, and the addition operation can be used.

This process achieves the decoupling of snoRNA-disease similarity and node degree. Firstly, $f_{s}$ **()** specifically explains the rationale for establishing a connection based on snoRNA-disease similarity. And $f_{d}$ **()** specifically explains the rationale for establishing a connection based on degree.

#### Optimization

The goal of this study is to reconstruct the snoRNA-disease network using the decoder under two conditions, represented by the adjacency matrix ***A***, with the corresponding posterior probability formalized as Equation 14:

$$p\left(A|Z\right)=\prod_{a}\prod_{b}p\left(A_{a,b}|z_{a},z_{b}\right),$$ (14)

By integrating the embedding loss values for snoRNA-disease similarity and node degree, the following can be derived as Equation 15:

$$L\left(\varphi ,\psi ;A\right)=E_{q_{\varphi }\left(Z^{s,d}\left|A,X\right.\right)}\left[\log\;p_{\psi }\left(\left.A\right|Z^{s,d}\right)\right]-\text{KL}\left(q_{\varphi }\left(Z^{d}\left|I,A\right.\right)\|p\left(Z^{d}\right)\right)-\text{KL}\left(q_{\varphi }\left(Z^{s}\left|X,A\right.\right)\|p\left(Z^{s}\right)\right),$$ (15)

were $Z^{s,d}$ represents the node embedding matrix derived for snoRNA-disease similarity and node degree. These are SDAs reconstructed under different conditions. ***KL()*** denotes KL divergence, a distance metric. Both $p\left(Z^{s}\right)$ and $p\left(Z^{d}\right)$ follow Gaussian priors $p\left(z_{a}\right)=N\left(z_{a}\left|0,I\right.\right)$ , where ***I*** denotes identity matrix. And $q\left(Z^{s}\right)$ and $q\left(Z^{d}\right)$ follow the posterior estimates of the Gaussian distribution, with $q\left(z_{a}|x_{a},A\right)$ following. Notably, In the case of node degree, the focus is on the node itself rather than the feature vector ***X***.

The first term of the above optimization objective can be further decomposed as follows:

$$E_{q_{\varphi }\left(Z^{s,d}\left|A,X\right.\right)}\left[\log\;p_{\psi }^{s}\left(\left.A\right|Z^{s}\right)\right]p^{s}+E_{q_{\varphi }\left(Z^{s,d}\left|A,X\right.\right)}\left[\log\;p_{\psi }^{d}\left(\left.A\right|Z^{d}\right)\right]q^{s},$$ (16)

where $p_{\psi }^{s}\left(\left.A\right|Z^{s}\right)$ and $\log\;p_{\psi }^{d}\left(\left.A\right|Z^{d}\right)$ follow Equations 12, 13, respectively, representing similarity and node degree. And $p^{s}$ and $q^{s}=1-p^{s}$ are Bernoulli parameters, representing the probabilities of establishing SDAs based on snoRNA-disease similarity and node degree, respectively. Finally, the corresponding parameters are learned using the EM algorithm.

## Results

### Experimental setup

This study evaluated the performance of the proposed DK-SDA model and comparison models using the RNADisease (Chen et al., 2023) and ncRPheo (Zhang W. et al., 2020) datasets. Six GNN-based comparison models were selected: NIMCGCN (Li et al., 2020), AMHMDA (Ning et al., 2023), NSAMDA (Zhao et al., 2022), iPiDA-GCN (Hou et al., 2022), VGAMF (Ding et al., 2021), and IGCNSDA (Hu et al., 2024). The NIMCGCN model was originally developed for miRNA-disease association prediction. Two GCNs were used to extract features from miRNA and disease similarity networks, followed by matrix completion to identify potential MDAs. Similarly, the AMHMDA model used two GCNs to extract features from miRNA and disease similarity networks, predicting potential MDAs via hypergraphs and hierarchical attention mechanisms. The NSAMDA model employed nearest neighbor and graph attention network (GAT) techniques to extract features from the miRNA-disease heterogeneous graph, predicting unknown MDAs via an inner product decoder. The VGAMF model employs VGAE technology to extract features from miRNA and disease similarity networks, using matrix decomposition to predict potential MDAs. The iPiDA-GCN model uses GCN to identify unknown piRNA-disease associations. The IGCNSDA model employs graph collaborative filtering to predict potential SDAs. NIMCGCN, AMHMDA, NSAMDA, and VGAMF were originally designed for MDA prediction, while iPiDA-GCN was developed for piRNA-disease association prediction. In this study, the inputs for these methods were changed to the RNADisease and ncRPheo datasets. All models used the same training, test, and external test sets. As in previous studies (Wei et al., 2024d; Zhang et al., 2024b), we primarily used AUC, AUPR, Accuracy (ACC), Precision (PRE), Sensitivity (SEN), and F1-Score (F1) metrics to evaluate model performance.

Our strategy manages the imbalance between positive and negative samples during model training. Specifically, we use a technique to randomly select an equal number of negative samples to match the positive samples in each training batch. This method ensures the model trains on a balanced dataset, crucial for preventing bias towards predicting non-interacting classes. Additionally, to further mitigate class imbalance risk, we employ stratified sampling during the cross-validation process. This strategy maintains a balanced distribution of positive and negative classes across different folds, enhancing the model’s generalizability and robustness.

### Performance comparison

Table 1 presents the results of all models on the RNADisease database. It is evident that the AMHMDA, NIMCGCN, NSAMDA, and VGAMF models perform poorly, likely due to their reliance on feature extraction from similarity networks. Determining an appropriate threshold for similarity networks is challenging, often resulting in the extraction of biased features. Comparatively, the performance of the NSAMDA and VGAMF models shows slight improvement. This improvement may be attributed to the use of nearest neighbor and GAT technologies in the NSAMDA model. The VGAMF model employs VGAE technology, which is better suited for SDA prediction tasks. The iPiDA_GCN and IGCNSDA models directly perform SDA prediction on the snoRNA-disease graph, leading to further performance improvements. Notably, the proposed model achieved 95.84% AUC, 97.23% AUPR, and 92.01% ACC, significantly outperforming all competing models. This superior performance may result from the proposed model’s use of the VGAE framework with decoupling technology, which mitigates the dominance of high-degree nodes in message propagation.

**TABLE 1:** Results of all models on the RNADisease dataset (%).

Models/Metrics	AUC	AUPR	ACC
AMHMDA	65.72	72.44	65.06
NIMCGCN	68.03	66.55	61.41
NSAMDA	70.23	75.88	68.26
VGAMF	73.04	76.65	68.09
iPiDA_GCN	81.01	81.73	70.54
IGCNSDA	84.38	87.44	78.31
Ours	95.84	97.23	92.01

Additionally, the proposed model incorporates KAN technology to enhance its flexibility and adaptability. Additionally, we tested the models’ adaptability to new data and evaluated their performance on external datasets. Specifically, all models were trained on the complete RNADisease dataset and subsequently tested on the external ncRPheo dataset. Table 2 presents the independent testing results of all models on external datasets. The results show that the proposed model significantly outperforms all competing models, except in the AUPR metric. These results demonstrate that the proposed DK-SDA model exhibits strong adaptability to new data.

**TABLE 2:** Results of all models on external testing set (%).

Models/Metrics	AUC	AUPR	ACC
AMNMDA	50.10	49.45	48.97
NIMCGCN	70.84	77.81	52.05
NSAMDA	50.72	67.26	69.11
VGAMF	70.69	70.49	64.57
iPiDA_GCN	61.82	61.85	66.51
IGCNSDA	71.42	87.46	72.90
Ours	80.33	81.92	75.17

### Performance evaluation

A 5-fold cross-validation experiment was conducted to further evaluate the model’s performance and minimize the impact of random factors. Table 3 presents the 5-fold cross-validation results of the proposed model on the RNADisease database. The proposed DK-SDA model performs well, achieving an average of 95.84% AUC, 97.23% AUPR, 92.01% ACC, 93.65% PRE, 90.14% SEN, and 91.86% F1. Additionally, the results for each fold are relatively stable, exhibiting small fluctuations. This further demonstrates that the proposed DK-SDA model exhibits strong adaptability and reliability.

**TABLE 3:** Results of 5-fold cross validation of DK-SDA model on RNADisease dataset (%).

Test set	AUC	AUPR	ACC	PRE	SEN	F1
1	94.12	96.28	90.41	91.94	88.58	90.23
2	95.19	96.87	92.01	93.40	90.41	91.88
3	95.73	97.18	92.24	93.84	90.41	92.09
4	96.94	97.80	92.69	94.31	90.87	92.56
5	97.20	98.03	92.69	94.74	90.41	92.52
Average	95.84 ± 1.27	97.23 ± 0.71	92.01 ± 0.94	93.65 ± 1.08	90.14 ± 0.89	91.86 ± 0.95

### Parameter experiments

**FIGURE 2:**
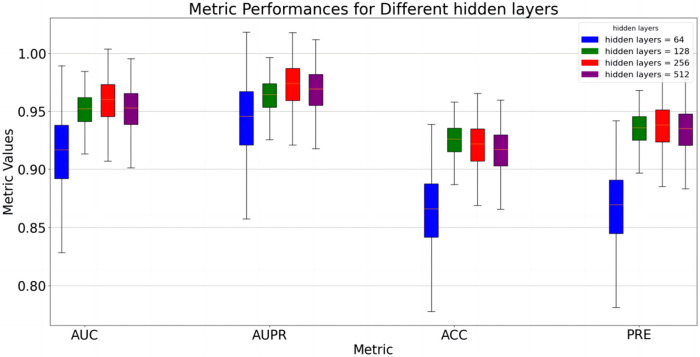
Model performance at different hidden layer dimensions.

Several sets of parameter experiments were conducted to evaluate the impact of parameter changes on the performance of the proposed model. First, the impact of varying hidden layer dimensions on model performance was tested. The hidden layer refers to the linear mapping layer in Equation 1, where the mapped features are used to generate regularized feature vectors. In the experiment, all other variables were held constant, with only the hidden layer dimension changed. The results are presented in Figure 2. The results indicate that when the hidden layer dimension is within the range [64,256], model performance gradually improves. When the hidden layer dimension exceeds 256, model performance slightly decreases. This may occur because smaller dimensions cannot fully extract features, while larger dimensions may cause information redundancy, limiting model performance. Therefore, a compromise hidden layer dimension can be selected to ensure the model’s adaptability to new data.

**FIGURE 3:**
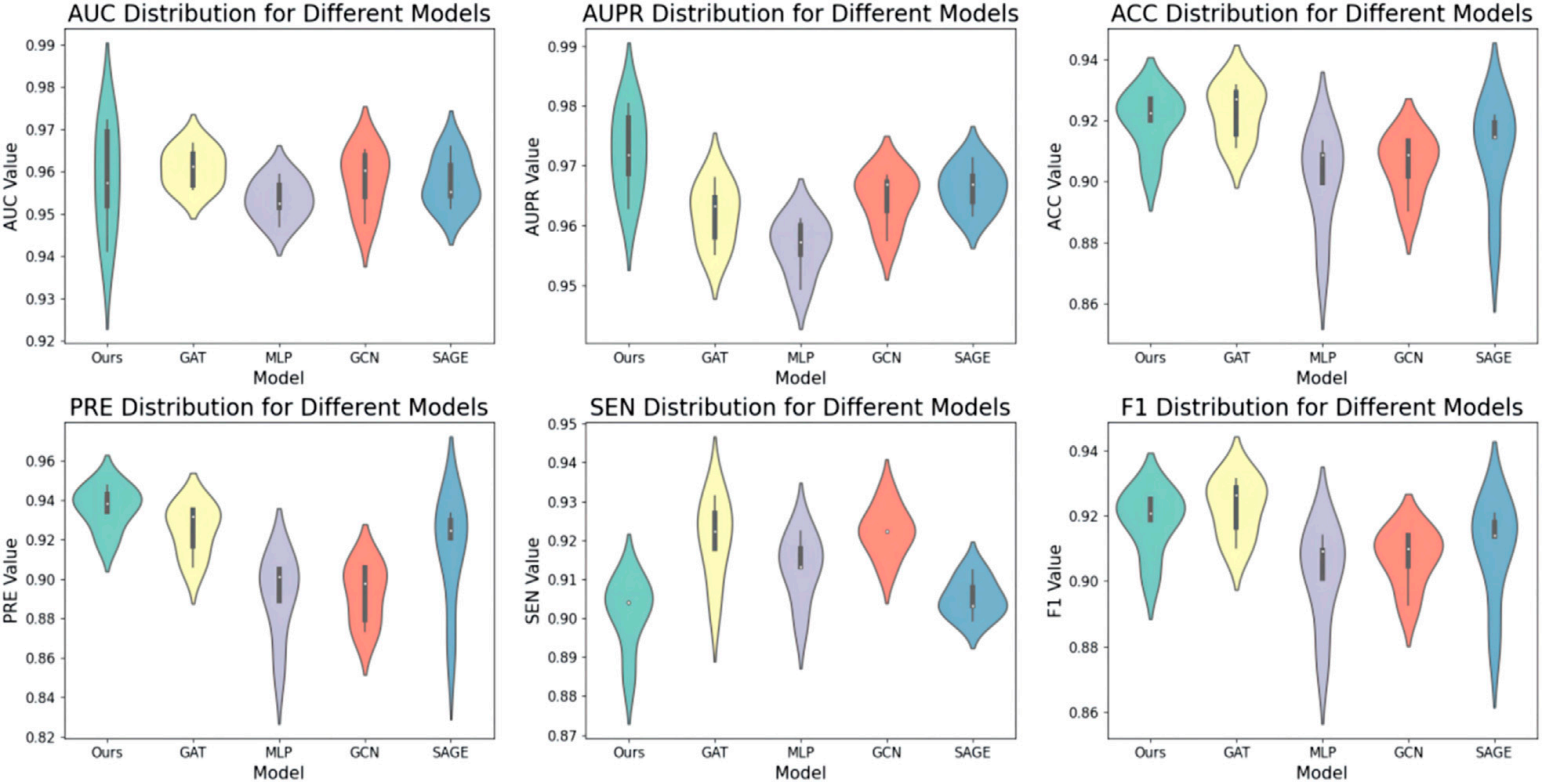
Results of the model with different encoders.

Secondly, we evaluated the impact of various encoders on model performance. The encoder is positioned in the second feature extraction layer of the node degree-based structure. First, the model applies a linear mapping layer to transform input features, followed by feeding them into the second feature extraction layer. We explore the model’s performance with different encoders in the second feature extraction layer, including KAN, MLP, GCN, GAT, SAGE, and GIN. In the experiment, all other variables were held constant, with only the encoder being modified. The results are presented in Figure 3. The results indicate that the model performs well when KAN is used as the encoder. Overall, the model’s performance decreases when using GNN, suggesting that high-degree nodes may dominate the message propagation process.

### Statistical significance analysis

**FIGURE 4:**
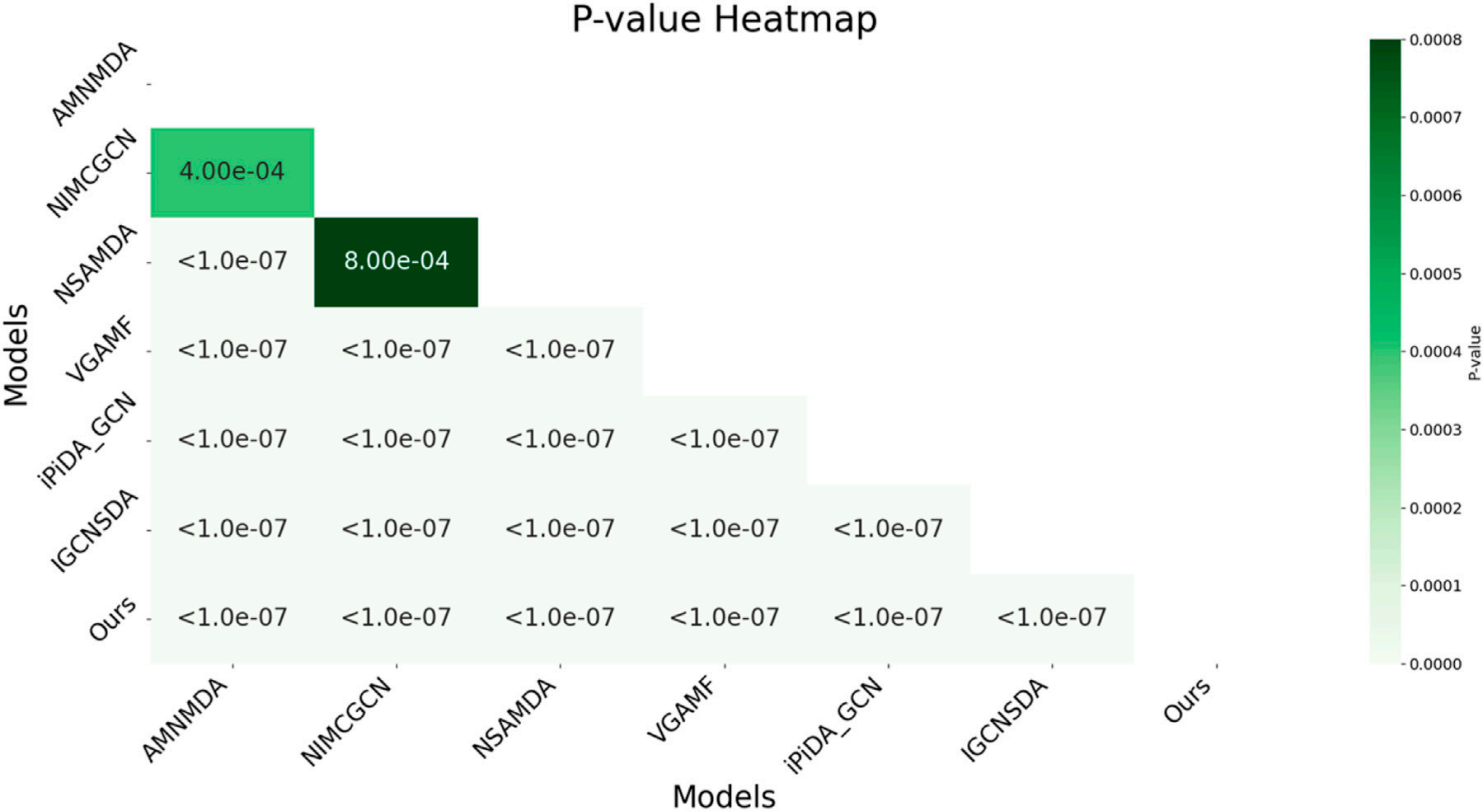
Statistical significance analysis of multiple methods based on AUC.

In this study, we employed the one-way analysis of variance (ANOVA) technique (St and Wold, 1989) to systematically compare the AUC performance of various snoRNA target prediction models on the snoRNA-disease association dataset, as shown in Figure 4. The results indicated that the proposed model was significantly superior on the dataset, achieving a p-value as low as 1.0e-07 when compared to most competing models, demonstrating its high statistical significance. Overall, the p-values between the model and all reference models were well below the significance threshold of 0.05, confirming its superior performance. This analysis not only underscored the model’s superior performance but also highlighted its stability and reliability across diverse datasets. Importantly, the findings provide robust evidence supporting the use of decoupled representation learning and KAN technology to enhance model generalizability.

### Case analysis

This study focuses on lung cancer, examining snoRNAs SNORD33 and SNORD78 through three case studies to evaluate the model’s performance in realistic scenarios. Lung cancer, a prevalent and serious disease, has high morbidity and mortality rates. Factors such as environmental pollution, smoking, and genetic mutations contribute to the onset and progression of lung cancer. Dysregulation of snoRNA *in vivo* can lead to tumorigenesis, manifested by cancer cell proliferation and altered gene expression. For instance, abnormal expression of SNORD78 has been linked to the initiation and progression of lung cancer (Zheng et al., 2015). Research indicates that inhibiting SNORD78 expression can curb cancer cell proliferation, suggesting its potential as a target for lung cancer therapy. Investigating lung cancer-related snoRNAs is likely to advance the development of novel therapeutic strategies.

SNORD33, typically located in the nucleolus, primarily facilitates the chemical modification of rRNA, such as guiding rRNA methylation (Mannoor et al., 2012). Dysregulation of SNORD33, whether through overexpression or underexpression, may contribute to cancer or inflammation development. Research has demonstrated that SNORD33 dysregulation disrupts rRNA methylation, resulting in abnormal protein complex synthesis. This disruption aids the growth and survival of lung cancer cells. Furthermore, SNORD33 dysregulation may facilitate cell metastasis and hasten lung cancer progression (Zhang et al., 2023). Similarly, SNORD78, a snoRNA with functions akin to SNORD33, promotes lung cancer cell growth, thus accelerating lung cancer progression.

In the first case study, we excluded all snoRNAs associated with lung cancer from the training set, and trained the model on the remaining data. Subsequently, the trained model predicted the likelihood of association between lung cancer and all snoRNAs, selecting the top 20 for further analysis. The results are detailed in Table 4. 18 of the predicted snoRNAs were validated using the RNADisease v4.0 database (Chen et al., 2023). Although SNORA21 has not been validated in the database, multiple studies suggest a strong association with lung cancer (Li L. et al., 2024). In the second and third case studies, we employed a similar approach to assess the association of SNORD33 and SNORD78 with various diseases, selecting the top 10 diseases for each. Results are presented in Tables 5, 6. For SNORD33, eight predicted diseases, excluding Prostate Neoplasm and Gliomas, were validated in the RNADisease v4.0 database (Chen et al., 2023). Similarly, for SNORD78, eight predicted diseases, excluding Osteosarcoma and Melanoma, were confirmed in the RNADisease v4.0 database. Additionally, in the second and third case studies, both SNORD33 and SNORD78 appear to be associated with lung cancer and related diseases. Furthermore, integrating findings from immune escape mechanism research can aid the development and optimization of lung cancer immunotherapies (Li et al., 2023). These results demonstrate the suitability of the proposed DK-SDA method for snoRNA therapeutic target prediction, facilitating the exploration of disease pathogenesis and the development of new treatment strategies.

**TABLE 4:** Top 20 predicted snoRNAs with potential associations with lung cancer.

snoRNA	RNADisease	snoRNA	RNADisease
SNORD28	Confirmed	SNORD14D	Confirmed
SNORD88B	Confirmed	SNORD18B	Confirmed
SNORD11B	Confirmed	SNORD113-8	Confirmed
SNORD112	Confirmed	SNORD78	Confirmed
SNORD46	Confirmed	SNORD114-12	Confirmed
SNORD104	Confirmed	SNORD51	Confirmed
SNORD54	Confirmed	SNORD103A	Confirmed
SNORD78	Confirmed	SNORD1C	Confirmed
SNORD76	Confirmed	SNORA21	Unconfirmed
SNORD72	Confirmed	SNORD21	Unconfirmed

**TABLE 5:** Top 10 predicted diseases with potential associations with SNORD33.

Disease	RNADisease	Disease	RNADisease
Lung Non-Small Cell Carcinoma	Confirmed	Lung Cancer	Confirmed
Lung Carcinoma	Confirmed	Colorectal Cancer	Confirmed
Gastric Cancer	Confirmed	Rheumatoid Arthritis	Confirmed
Traumatic Brain Injury	Confirmed	Prostate Neoplasm	Unconfirmed
Prostate Cancer	Confirmed	Glioma	Unconfirmed

**TABLE 6:** Top 10 predicted diseases with potential associations with SNORD78.

Disease	RNADisease	Disease	RNADisease
Lung Non-Small Cell Carcinoma	Confirmed	Colorectal Cancer	Confirmed
Lung Cancer	Confirmed	Prostate Cancer	Confirmed
Clear Cell Renal Cell Carcinoma	Confirmed	Gastric Cancer	Confirmed
Hepatocellular Carcinoma	Confirmed	Osteosarcoma	Unconfirmed
Head And Neck Squamous Cell Carcinoma	Confirmed	Melanoma	Unconfirmed

## Conclusion

SnoRNA dysregulation significantly contributes to disease onset and progression, making accurate identification of snoRNA therapeutic targets essential for redevelopment of traditional medicines. Timely identification of potential snoRNA therapeutic targets facilitates early disease screening and the development of novel treatment strategies. Existing GNN-based deep learning methods reveal network topology and accurately predict potential snoRNA therapeutic targets. However, these methods encounter significant challenges. Datasets are often imbalanced due to limitations in data collection. During message propagation in GNNs, high-degree nodes often dominate, hindering accurate node representation learning. To address these challenges, this study proposes an effective snoRNA therapeutic target prediction model within the VGAE framework, integrating decoupling and KAN techniques. The model learns snoRNA and disease representations within the VGAE framework, reconstructing the snoRNA-disease graph to identify potential therapeutic targets. The decoupling technique separates similarity from node degree, mitigating the dominance of high-degree nodes in information propagation and enhancing the accuracy of snoRNA therapeutic target prediction, particularly for diseases like lung cancer. Additionally, the model integrates KAN technology to enhance flexibility and adaptability to new data. Moreover, the model indicates that snoRNA SNORA21, SNORD78 and SNORD33 may play a critical role in lung cancer. Experimental results demonstrate the model’s high efficiency in disease research, particularly in lung cancer studies, providing valuable references for early screening and the redevelopment of traditional medicines.

This study integrates the decoupling of node similarity and node degree with KAN technology within the VGAE architecture, enhancing the accuracy of snoRNA therapeutic target predictions. However, there are several potential limitations in this study. First, KAN technology has high computational complexity. Future work will focus on exploring parallel designs for KAN. Secondly, the proposed model does not account for the sequence, structure, function, and other biological data of snoRNA, nor does it include clinical information on the disease and specific targets, which limits the model’s performance. To address this, we plan to integrate large language models in the future to extract and combine multimodal data, enhancing the discovery of potential snoRNA therapeutic targets. Additionally, the model faces challenges when encountering snoRNA targets that deviate from established topological patterns or are previously unobserved. To improve the model’s adaptability to out-of-distribution data and capture interaction patterns differing from traditional topological structures, methods such as transfer learning and meta-learning can be employed.

## Data Availability

The original contributions presented in the study are included in the article/supplementary material, further inquiries can be directed to the corresponding authors.
